# Salter Harris Fractures of the Distal Femur: Learning Points From Two Cases Compared

**DOI:** 10.1177/2324709613500238

**Published:** 2013-07-29

**Authors:** Sean M. McKenna, Steven W. Hamilton, Simon L. Barker

**Affiliations:** 1 Royal Aberdeen Children’s Hospital, Foresterhill, Scotland

**Keywords:** Salter Harris distal femoral fracture, knee dislocation, vascular injury

## Abstract

Salter Harris–type injuries of the distal femur should be treated as a dislocation of the knee and therefore as a medical emergency. Senior medical staff should be involved early, ankle–brachial index ratio should be measured in all patients and the clinician should have a high index of suspicion for a vascular injury. Ideally reduction, stabilization, and vascular repair, if necessary, should be carried out within 6 hours of the initial event. There should be a low threshold for fasciotomies. These 2 cases demonstrate the importance of having a high index of suspicion for vascular injury and the need for continued reassessment.

The distal femoral physis is responsible for approximately 70% of the growth of the femur and 35% of the total length of the lower extremity. It has an average growth of 1.0 cm/year, which makes it the fastest growing physis.^1-3^

Compared with the ligamentous structures, the physis is generally considered the weaker link within joints of children, and therefore most periarticular injuries have involved the growth plates.^4^

Distal femoral physeal fractures can have a high incidence of long-term complications, such as growth disturbance, with subsequent development of leg length discrepancy and/or angular deformities.^5^ The potential for complications is related to the velocity of the injury as well as the age of the child at injury.

## Case 1

**Figure 1.**
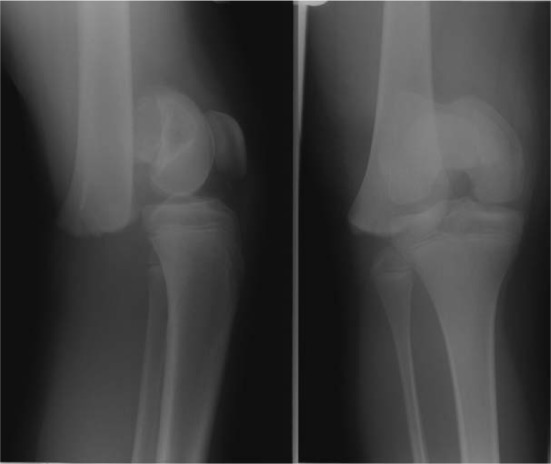
AP and lateral radiographs showing SH-1 fracture of the distal femur.

Patient 1, aged 11 years and 10 months, sustained a closed Salter Harris (SH) 1 fracture of her distal femur after a hyperextension injury on a trampoline (Figure 1). Popliteal and distal pulses were present, and there was no neurological deficit.

**Figure 2.**
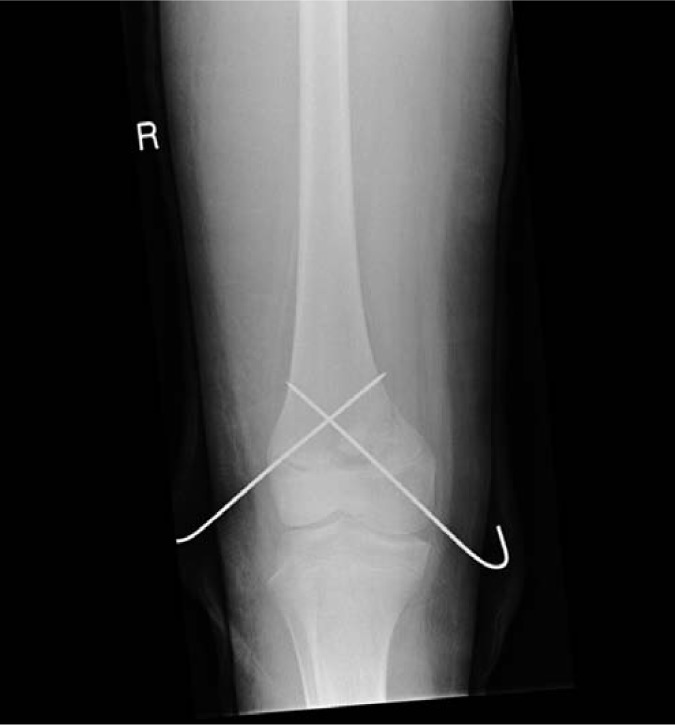
Postoperative AP radiograph showing reduction and Kirschner-wire fixation.

She was treated as per Advanced Trauma Life Support (ATLS) protocols and proceeded to theatre for urgent closed reduction and internal fixation (Figure 2).

**Figure 3.**
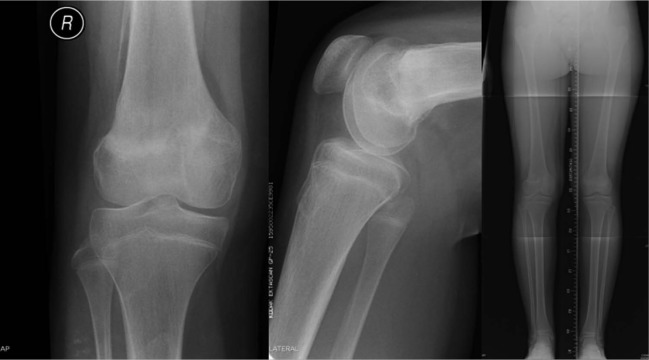
Radiographs at 1 year demonstrating satisfactory reduction and leg length.

She recovered well and went on to have normal function and no limb length discrepancy (Figure 3). This may have been contributed to by the fact that she had reached menarche and was reaching skeletal maturity.

## Case 2

**Figure 4.**
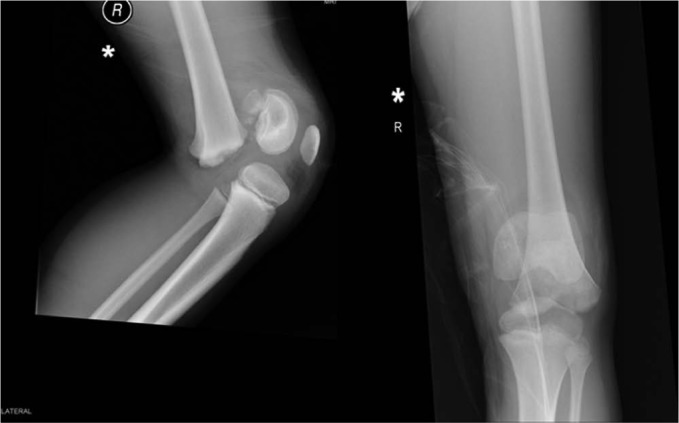
AP and lateral radiographs showing SH-2 injury of the distal femur.

Patient 2, aged 7 years and 9 months, sustained an SH type-2 fracture of his distal femur after a hyperextension injury when he fell off a wall (Figure 4).

This was a closed injury and again popliteal and distal pulses were present. He was treated via ATLS protocols, but while waiting for his surgery his distal pulses became weak.

**Figure 5.**
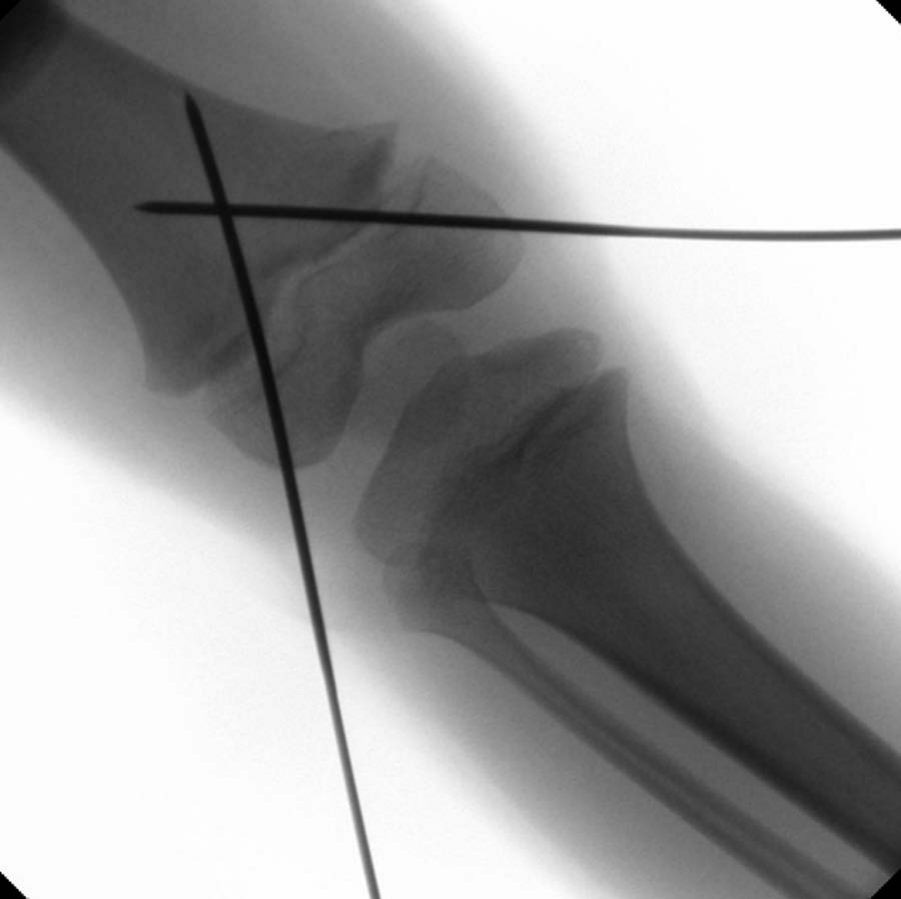
AP radiograph of the distal femur with satisfactory reduction and fixation with Kirschner wires.

**Figure 6.**
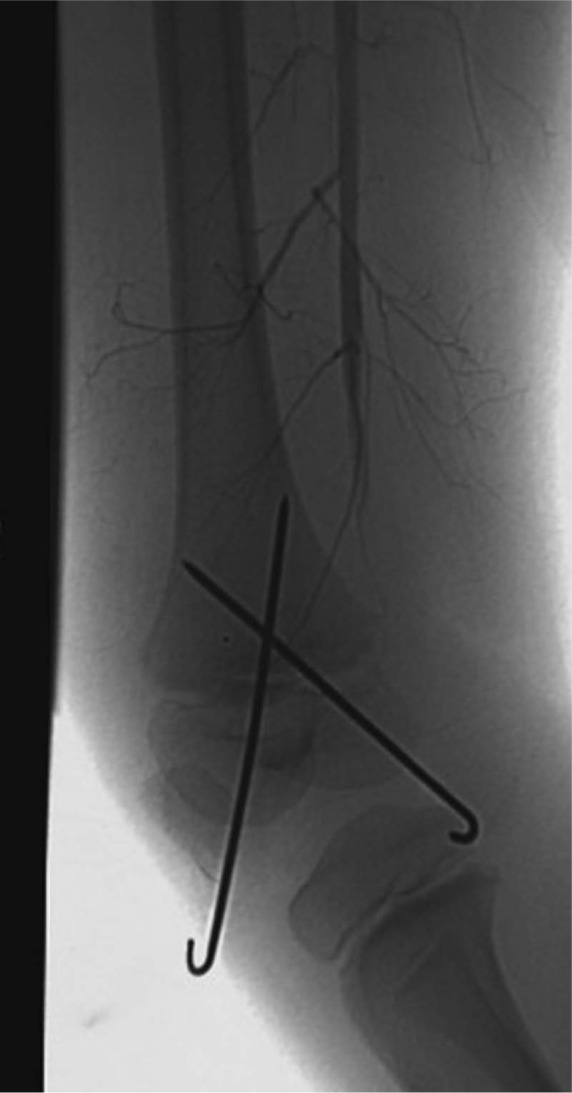
On-table angiography demonstrating occlusion of the popliteal artery and no distal run-off.

He proceeded to urgent reduction and internal fixation (Figure 5), and subsequent angiography revealed the suspected popliteal injury (Figure 6).

He required a venous interposition graft and fasciotomies to prevent compartment secondary to a reperfusion injury. Nevertheless, he lost anterior compartment muscle mass suggesting circulatory compromise may have been more prolonged than initially suspected.

**Figure 7.**
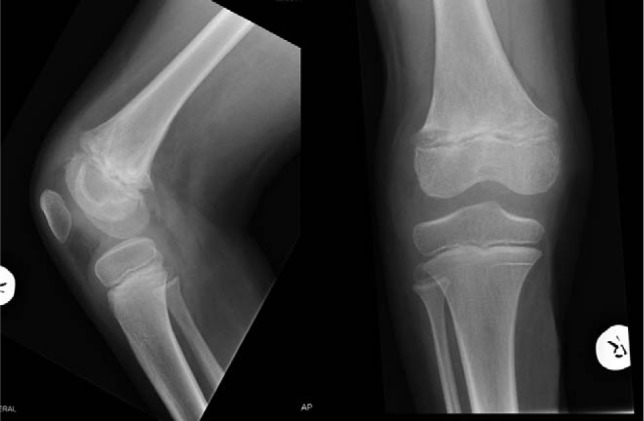
AP and lateral radiographs at 1 year showing no skeletal abnormality.

At his follow-up he continues to have a good level of function and thus far has limited growth disturbance in the form of a leg length discrepancy with a mild foot drop (Figure 7). He has several years of growth remaining and therefore will continue to be followed-up until he reached skeletal maturity.

These injuries frequently occur during sporting activities. This is usually a hyperextension injury with anterior displacement of the epiphysis; subsequently there is a significant risk of vascular complications to the popliteal vessels. The possibility of an associated vascular injury has been reported at almost 20% in some studies, and this can lead to significant rates of limb loss, functional disability, and mortality.^6,7^

Although clinically this diagnosis can be obvious, it is important to remember that a percentage of these cases can present spontaneously reduced so the clinician must be suspicious of the possibility of this injury. For those cases that present displaced, priorities include early reduction and splintage initially. One of the most important screening tools for a vascular injury is ankle–brachial index ratio (ABPI), which is a necessity if this injury is suspected.

If either ABPIs or clinical examination suggest a vascular injury, then formal imaging techniques should be carried out and repair of any defect to revascularize the distal limb must be carried out as soon as possible and certainly within 6 hours of the initial injury. Although angiography is recommended, it must not delay transport to a trauma hospital for revascularization, and in some cases these injuries can be treated effectively without angiography before surgery.^6,8^

This obviously necessitates a number of different specialties working together quickly and decisively to provide adequate care. Senior medical staff should be involved from early in the patient presentation.

SH-type injuries of the distal femur should be treated as a dislocation of the knee and therefore as a medical emergency. Senior medical staff should be involved early, ABPIs should be measured in all patients, and the clinician should have a high index of suspicion for a vascular injury. Ideally reduction, stabilization, and vascular repair, if necessary, should be carried out within 6 hours of the initial event. There should be a low threshold for fasciotomies. These 2 cases demonstrate the importance of having a high index of suspicion for vascular injury and the need for continued reassessment.
